# Supplementary material for: CT characteristics and diagnostic value of COVID-19 in pregnancy
Source: PLoS One. 2020 Jul 2;15(7):e0235134. doi: 10.1371/journal.pone.0235134 (PMC7331988; doi:10.1371/journal.pone.0235134)
Supplement: Attachment — Submitted filename: comments.docx [file pone.0235134.s001.docx]

**Review:** “CT characteristics and diagnostic value of COVID-19 in pregnancy”

**General comments:**
This is a research article involving 10 pregnant women with COVID-19 in Wuhan from January 20, 2020 to February 6, 2020. Among them, 6 patients were laboratory- confirmed and 4 clinically-diagnosed cases. COVID-19 is a rapidly evolving pandemic. Pregnant woman is a special population. Limited data are available about COVID-19 during pregnancy. The understanding of clinical and radiological features of COVID-19 during pregnancy is important for the outcomes of mothers and fetuses.

This study retrospectively described the CT characteristics and assessed the diagnostic value for COVID-19 in pregnancy, which could help to interpret COVID-19 during pregnancy. I recommend a publication of this paper with major revisions.

**Abstract**:

1. Methods: “Clinical and chest CT data were collected and clinical symptoms, laboratory indicators, and CT images were analyzed to explore CT characteristics and diagnostic value of pregnancy with COVID-19”. The “…of pregnancy with COVID-19” should be written as“… for COVID-19 during pregnancy.”

Clinical symptoms and laboratory indicators were also analyzed. Were there any typical or atypical features need to be presented in Results?

2. Results: GGO: abbreviation should not be used for the first presentation.

3. Results: “Dynamic observation was performed in 4 cases after treatment”. Three cases showed progression or improvement, what was the follow-up time when progression or improvement occurred?

4. Conclusions: “The CT characteristics of COVID-19 in pregnancy are mainly in early and progressive stages”. What are the CT characteristics you are supposed to summarize for COVID-19 diagnosis?

**Key words:** OK

**Introduction:**

1. “Pregnant women are also susceptible population, and they are more likely to have complications than others, and even progress to severe cases.” Do you mean the pregnant women with COVID-19 are more likely to have complications or other concomitant pathogens? Do you have some references?

2. “However, COVID-19 in pregnancy is often found to be associated with pleural effusion.” Why? Do you have references? Is the pleural effusion possible to be intrinsically related with the pregnant status?

3. The introduction is too simple to state the current studies of COVID-19.

**Materials and Methods:**

**1. 1 Patients**

(1) “Patients an clinical and laboratory findings”. Please revise it.

(2) Did the Institutional Ethics Committee approve the study?

(3) “Among these 10 pregnant women, 6 tested positive for novel coronavirus nucleic acid”. How about the sample and test methods? Use throat swab samples and RT-PCR test?

(4) “Their age ranges from 26 to 40 year old, with an average age of 30.” The sample size was small, were the age, gestational age, and onset time in normal distribution? If not, median data were more appropriate.

**1.2 Examination method**

(1) Did all the pregnant preform CT scans before delivery?

(2) The thickness and interval was 1.0mm? How about the CT dose?

(3) Image interpretation can be described in another paragraph.

(4) Were there any low dose CT technologies implemented in the present study?

**Results:**

1. Distribution: Did you analyze the peripheral or non-peripheral distribution?

2. GGO: abbreviation should not be used for the first presentation.

3. “Other radiographic signs were also found that there were 6(60%) cases with small bilateral pleural effusions (figure 3) and no case with lymphadenopathy.” Need to be revised for avoiding the confusion. What were the diagnostic criteria for lymphadenopathy? Please add the criteria in “Materials and Methods” part.

**Discussion:**

1. Please state the main findings of your study in the first paragraph.

2. “6/10(60%) cases have small bilateral pleural effusion, which is not in line with reports that pleural effusion is rare according to Diagnosis and treatment of pneumonia for novel coronavirus infection (trial version 5)”. Please check whether the pleural effusion is associated with COVID-19 or pregnant status.

3. “all the 10 patients were in the early and progressive stages, and there was no critical stage; 6 cases (60%) were at early stage based on CT images: 2 cases (20%) showed single small patchy GGO in one lobe, and 4 cases (40%) showed multiple patchy GGO in the periphery of both lungs. Among them one case was normal in the first CT scan, which was consistent with the atypical COVID-19 performance reported by Chung M et al. [12]. The other 4 cases (40%) were at progressive stage, showing multiple small or large patchy GGO in both lungs. Some lesions were consolidated with sign of intra-bronchial air-bronchogram.” How to define the progressive stage? Peripheral distribution is common for COVID-19; however, the distribution was not demonstrated in the abstract or results.

4. Except for the pleural effusion, were there other CT characteristics for COVID-19 in pregnancy, which could help for the diagnosis?

5. Limitation part should be restructured before conclusion.

Please re-edit the language from abstract to discussion.

**References**: Please update some epub ahead of print references.
